# Determining initial and follow-up costs of cardiovascular events in a US managed care population

**DOI:** 10.1186/1471-2261-11-11

**Published:** 2011-03-16

**Authors:** Richard H Chapman, Larry Z Liu, Prafulla G Girase, Robert J Straka

**Affiliations:** 1 US Health Economics & Outcomes Research, IMS Health, Falls Church, VA, USA; 2 Pfizer Inc., New York, NY, USA; 3 Weill Cornell Medical College, New York, NY, USA; 4 University of Minnesota College of Pharmacy, Minneapolis, MN, USA

## Abstract

**Background:**

Cardiovascular (CV) events are prevalent and expensive worldwide both in terms of direct medical costs at the time of the event and follow-up healthcare after the event. This study aims to determine initial and follow-up costs for cardiovascular (CV) events in US managed care enrollees and to compare to healthcare costs for matched patients without CV events.

**Methods:**

A 5.5-year retrospective matched cohort analysis of claims records for adult enrollees in ~90 US health plans. Patients hospitalized for first CV event were identified from a database containing a representative sample of the commercially-insured US population. The CV-event group (n = 29,688) was matched to a control group with similar demographics but no claims for CV-related events. Endpoints were total direct medical costs for inpatient and outpatient services and pharmacy (paid insurance amount).

**Results:**

Overall, mean initial inpatient costs were US dollars ($) 16,981 per case (standard deviation [SD] = $20,474), ranging from $6,699 for a transient ischemic attack (mean length of stay [LOS] = 3.7 days) to $56,024 for a coronary artery bypass graft (CABG) (mean LOS = 9.2 days). Overall mean health-care cost during 1-year follow-up was $16,582 (SD = $34,425), an excess of $13,792 over the mean cost of matched controls. This difference in average costs between CV-event and matched-control subjects was $20,862 and $26,014 after two and three years of follow-up. Mean overall inpatient costs for second events were similar to those for first events ($17,705/case; SD = $22,703). The multivariable regression model adjusting for demographic and clinical characteristics indicated that the presence of a CV event was positively associated with total follow-up costs (P < 0.0001).

**Conclusions:**

Initial hospitalization and follow-up costs vary widely by type of CV event. The 1-year follow-up costs for CV events were almost as high as the initial hospitalization costs, but much higher for 2- and 3-year follow-up.

## Background

Cardiovascular (CV) events are prevalent and expensive worldwide both in terms of direct medical costs at the time of the event and follow-up healthcare after the event. In 2006, the average cost at discharge for a Medicare beneficiary with a principal diagnosis of CV disease (CVD) was $10,201 [1]. Continuing improvements in medical care make contemporary assessments of incremental CVD costs relevant and necessary for up-to-date economic analyses. In addition, it is important that health economic analyses consider the costs of specific CV events in addition to a broad assessment of CVD.

Given the lack of up-to-date cost estimates for specific CV events in the literature, we performed a retrospective, matched cohort analysis of transactional billing (claims) records to determine the cost of various specific CV events. This study assessed the differences in total direct medical costs for those with and without CV events while controlling for potential confounders and underlying differences that may exist among study groups as a means to assess the incremental cost of CV events over average health care costs. This study also examined cost differences between initial and subsequent CV events, as well as differences in costs among patients with comorbidities of hypertension (HTN), type 2 diabetes mellitus (DM), and dyslipidemia (DYS).

The primary study objectives were to: determine overall costs of initial and subsequent CV events in patients who have been hospitalized with a CV event; examine costs of 1-, 2-, and 3-year follow-up care among all patients with a CV event; and determine incremental costs for CV events by comparing total costs of follow-up care for patients with a CV event to similar patients without a CV event.

## Methods

### Ethical Approval

Request to include details of the Institutional Review Board that granted ethical approval or any requirements for permission to use the data sets.

### Data

Claims data were obtained from the IMS LifeLink: US Health Plan Claims data base (formerly called the PharMetrics Patient-Centric database), a nationally-representative longitudinal database containing fully adjudicated medical and pharmaceutical claims for >50 million unique patients participating in >90 commercial health plans across the US. The database includes: inpatient and outpatient diagnoses (as ICD-9-CM codes) and procedures (as CPT-4 and HCPCS codes), as well as retail and mail order prescription records. CV events and procedures of interest and the corresponding codes are shown in Table 1. Claims dates were available for all services rendered. Additional data include: demographic variables (age, gender, geographic region); product type (e.g., Health Maintenance Organization [HMO], Preferred Provider Organization [PPO]); payer type (e.g., commercial, self-pay); provider specialty; and start and stop dates for plan enrollment.

**Table 1:** Codes for CV events, co-morbid conditions and procedures

Diagnoses and Procedures	Codes
	
**Event**	**ICD-9-CM**
Heart failure with or without CKD	398.91, 402.01, 402.11, 402.91, 404.01, 404.03, 404.11, 404.13, 404.91, 404.93, 428.xx
Myocardial infarction	410.xx, 412
Unstable angina and angina pectoris	411.1, 413.x
Other ischemic heart disease	411.xx (except 411.1, 414.xx, 427.xx, V45.81, V45.82
Ischemic stroke	433.xx, 434.xx, 436, 437.0, 437.1, 438.xx, 997.02
TIAs and other CVAs	435.x
Peripheral vascular disease	440.0, 440.1, 440.2x, 443.xx
**Co-morbid conditions**	**ICD-9-CM**
Hypertension	401.xx, 401.0, 401.1, 401.9, 402.xx, 403.xx, 404.xx, 796.2
Dyslipidemia	272.xx
Diabetes mellitus	250.x0, 250.x2, 250.x
	
**Procedure**	**CPT-4**
CABG	33503 - 33545
Coronary stenting	92980, 92981
Percutaneous transluminal coronary angioplasty/thrombectomy/atherectomy	92973, 92982, 92984, 92995, 92996
Percutaneous transluminal pulmonary artery balloon angioplasty	92997, 92998
Carotid endarterectomy	35301, 35390, 35901

CKD, chronic kidney disease; TIA, transient ischemic attack; CVA, cerebrovascular accidents; CABG, coronary artery bypass graft

### Study Period

The 5.5 year study period (including a 1-year identification period) ranged from January 1, 2001 to June 30, 2006. All patients with an index hospitalization for a CV event were identified for analysis. For these patients, readmission could occur during the follow-up period (up to June 30, 2006).

Study data were divided into 3 periods: identification, pre-index, and follow-up. The identification period was from January 1, 2001 through June 30, 2005. The first hospitalization with relevant diagnosis or procedure codes for a CV event during this period was designated as that patient's index event. If a hospitalization was associated with >1 diagnosis or procedure code of interest, the patient was included in each relevant event type; i.e., event types were not mutually exclusive. For example, if a patient was admitted for MI and then underwent CABG, that patient would be included in both the MI and CABG categories. The date of first hospital discharge with a diagnosis or procedure code of interest served as the index date for that patient. The pre-index period was defined as 1 year (12 months) prior to index hospitalization admission date. Thus, beginning and ending dates of the pre-index period varied among patients with the earliest time at which the pre-index period could begin being January 2000. Three follow-up periods were used to create cohorts of patients with 12-, 24-, or 36-month continuous enrollment after discharge from the index hospitalization. Follow-up terminated at the end of the patient's enrollment period in the health plan or the study end date (June 30, 2006). Patients who died were included regardless of follow-up length. Because deaths are not available in the claims data (due to privacy concerns), a proxy algorithm was used to determine patient deaths; in brief, patients were assumed to have died if they had evidence of specific mortality-associated events during the last month in which medical and pharmacy claims were available prior to disenrollment. Thus, beginning and ending dates of the follow-up period varied among patients but could not extend beyond June 30, 2006.

Patients were tracked from admission for the index hospitalization (or index date for non-event patients) until the end of enrollment in the health plan or study end date, whichever came first. All records relating to the index hospitalization and subsequent follow-up from the index date were extracted for analysis. Data pertaining to the first CV event readmission in the follow-up period were also extracted for analyses.

### Patient Selection

Subjects were stratified into two cohorts based on presence or absence of hospitalization for a CV event. The CV-event group included all patients with a complete hospitalization (admission and live discharge within the identification period) containing relevant procedure or primary diagnosis codes for a CV event during the identification period. A matched control group was used to compare total follow-up costs between patients with and without CV events.

Exclusion criteria for both CV-event and control groups included: patient not continuously eligible for drug and health benefits during their entire pre-index and follow-up periods; health plan did not report days supplied or quantity dispensed for medications; patient <18 years of age at index; patient ≥65 years of age whose insurance coverage was not "Medicare Risk" at any time during study period (complete claims histories may not be available for patients without Medicare Risk coverage due to benefit coordination issues with other payers); patient with evidence of pre-existing CVD (claims for CV-related diagnoses or procedures in the pre-index period); or patient with index hospital stay of more than 27 days (99th percentile).

All records from the pre-index period, index event (first hospitalization for the CV-event group and matched index date for the non-event group), and follow-up from the index date (to the end of June 2006) were extracted. Records were linked using a unique patient code. All medical, laboratory, and pharmacy claims were compiled for the period January 1, 2000 to June 30, 2006.

**Figure 1:**
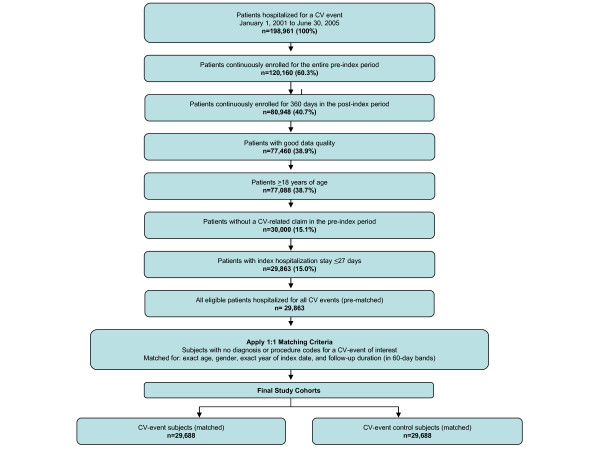
**Diagram of study cohort identification procedure**.

An index date was randomly assigned to control-pool subjects during their health plan enrollment which would guarantee collection of ≥1 year of pre- and post-index data. Control-pool subjects were then matched with CV-event subjects (on a 1:1 basis) by exact age, gender, exact year of index date, and follow-up duration (in 60-day bands). As the intent was to compare total costs of CV-event follow-up to costs for an average patient, and not to determine incremental costs of CV events *per se*, patients were not matched on comorbid conditions. A diagram of the study cohort identification procedure is shown in Figure 1. In cases where a CV-event subject had <12 months of follow-up data due to death, they were matched with a control subject who also had <12 months of follow-up. In these cases, the index date for the CV-event subject was assigned to the control subject. All unsuccessfully matched control-pool subjects were excluded from the matched analyses.

Subjects with a CV event were also evaluated for evidence of HTN, DYS, or DM during the pre-index period. Subsets of subjects with ≥1 of these three comorbidities of interest were created for analysis, with some subjects included in >1 subset. Subjects with HTN, DYS, or DM were identified by presence of relevant ICD-9 codes (Table 1). Diabetes was also identified by presence of any oral diabetes medication (with or without insulin).

For all CV-event subjects, we determined whether there were any subsequent CV events after the index hospitalization discharge. Subsequent events were determined by finding complete hospitalizations with relevant diagnosis or procedure codes of interest during the follow-up period. The number of subsequent events per subject and total medical costs associated with the first subsequent (ie. second) CV event were calculated.

### Measures of Interest

Measures of interest for analysis included: subject demographic and clinical characteristics; number of subsequent CV events; total medical costs following index CV event; direct medical costs associated with the index hospitalization (total cost and LOS); direct medical costs of hospitalization associated with second CV event hospitalization (total cost and LOS); total all-cause follow-up medical costs after index hospitalization discharge; total CV-related follow-up costs after index hospitalization discharge (ie. claims for outpatient CV medications or CV-related procedures).

Total medical costs included all healthcare costs whether CV- or other-related. Direct medical costs included any cost incurred during the inpatient hospitalization associated with the CV event, ie. including co-pay. If a CV event was the primary diagnosis in any hospitalization (index or readmission), all costs from that hospitalization were attributed to that CV event and used in calculating total direct cost. Costs were summed from the admit date to the discharge date for hospitalizations. Costs were calculated based on allowed health plan payments for medications and services and expressed in 2006 US dollars ($). Costs were also updated where necessary using the medical care component of the US Consumer Price Index.

### Statistics

Means, medians, and SDs for each cohort were calculated and reported for continuous variables. Distributions and frequencies were calculated for categorical variables. Overall subject characteristics were reported as well as for subject subsets (i.e., subjects with comorbid HTN, DYS, and/or DM).

Generalized linear models were used in main effects multivariable regression analyses of 1-year follow-up costs to account for any remaining differences in variables used to match subjects and non-normally-distributed payments (common when using healthcare claims data). For the CV-event group, the number of patients with a subsequent CV event in the follow-up period was assessed, as well as the number of subsequent CV events per person-year of follow-up. Total direct medical costs and hospital LOS associated with initial and subsequent CV events were determined. The rate of subsequent CV events and costs associated with initial and subsequent CV events are reported overall as well for subsets with comorbid HTN, DYS, or DM.

Because this was a non-controlled observational study, it was expected that some degree of bias would exist among study groups. Matching non-CV event subjects and adjustments in multivariable analyses were used to control for these biases. The following subject-specific covariates were adjusted for in multivariable analyses: age group at index date; gender; health plan type (consumer-directed health care, HMO, indemnity, point of service [POS], PPO, other/unknown); health plan payer type (commercial, Medicaid, Medicare risk, self-insured, other/unknown); geographic region at index date (Northeast, Midwest, South, West); physician specialty at discharge from CV event hospitalization (family practice/general practitioner [FP/GP], internal medicine, cardiologist, other, unknown); Dartmouth Manitoba version of Charlson Comorbidity Index [CCI]; presence of DYS; presence of statin therapy or other lipid-lowering therapy in the pre-index period; presence of HTN; presence of type 2 DM; presence of other medications to treat comorbidities of CV disease; total health-care costs (i.e., payments by health plans to providers) during the 12-month pre-index period. Only main effects were considered; no interaction effects were assessed in the models.

The excess cost of a CV event during follow-up was estimated by comparing the total cost of care for CV-event subjects to costs for control (non-CV-event) subjects during follow-up. Resource utilization and costs were calculated for the first, second, and third years of follow-up. For this analysis only, we required subjects to have at least 12-, 24-, or 36-months of post-index data, as needed. Utilization and costs are reported by type of service and overall in the following categories: medications (CV-related therapies, all other medications); outpatient care (emergency room visits, physician office visits, imaging tests, laboratory procedures, all other outpatient services); inpatient care (number of hospitalizations, hospital LOS in days).

Along with total health-care costs, CV-related costs were also calculated. A claim was categorized as CV-related if it was for diagnosis or procedure codes indicative of disease (as listed above), for statin therapy, or other lipid-lowering therapy, and for other concomitant CV medications.

All analyses were conducted using SAS^® ^version 8.02 (SAS Institute Inc., Cary, NC, USA).

## Results

Of the 198,961 patients identified with an index hospitalization for a CV event between January 1, 2001 and June 30, 2005, the majority were excluded, predominantly for not fulfilling continuous enrolment requirements or for having a CV-related claim in the pre-index period (Figure 1). A total of 29,688 individuals met criteria to be CV-event subjects and were matched with 29,688 control (non-CV-event) subjects. Among all (matched) CV-event subjects, mean (SD) age was 55.5 (12.0) years and 37.2% (11,097) were female. Baseline demographic and clinical characteristics for all matched subjects, as well as matched CV-event subjects and matched controls, are shown in Table 2.

**Table 2:** Baseline demographic and clinical characteristics

Demographic and Clinical Characteristics	CV-Event Patients (matched)	CV-Event Control Patients (matched)	P-value
Total Subjects	29,688	29,688	

Age, mean (years)	55.5	55.4	0.5474
Age groups, %			0.9963
18-34	2.55	2.53	
35-44	11.46	11.55	
45-54	34.72	34.74	
55-64	38.05	37.94	
65+	13.22	13.23	

Gender, %			1.00
Female	37.15	37.15	

Payer type at index, %			<0.0001
Commercial Plan	74.75	74.35	
Medicare	1.10	0.50	
Medicaid Risk	13.87	13.41	
Self-Insured	4.92	5.88	
Unknown	5.35	5.85	

Geographic Region at index, %			<0.0001
Northeast	19.26	22.31	
Midwest	42.58	41.34	
South	32.18	27.59	
West	5.98	8.76	

Duration of follow-up, mean (days)	696	696	0.7103

CCI, mean	0.56	0.27	<0.0001

Pre-index presence of DYS, %	30.12	22.08	<0.0001

Pre-index presence of HTN, %	39.01	22.01	<0.0001

Pre-index presence of DM, %	18.39	7.06	<0.0001

Pre-index 12-month total healthcare costs (mean), $ in 1000s	$5124	$2652	<0.0001

CV = cardiovascular; CCI = Charlson Comorbidity index; DYS = dyslipidemia; HTN = hypertension; DM = diabetes mellitus

Overall inpatient costs for index hospitalizations for CV-event subjects were $16,981 per case (SD = $20,474; median = $9996) with an average (SD) length of stay of 4.36 (3.06) days. These costs varied considerably by type of CV event and ranged from a mean of $6699 for TIA/other CVAs and $6910 for angina to $56,024 for CABG. Mean LOS varied in a similar manner, from 2.71 days per angina hospitalization to 9.23 days for CABG (Table 3).

**Table 3:** Unadjusted costs of index hospitalization among all CV event subjects

	Overall	CABG	Other CV Procedures	MI	Heart Failure	Angina	Other Ischemic Heart Disease	Stroke	TIA/other CVAs	PVD
**Total patients **n	29,688	1894	8581	9151	2230	1650	11,224	2600	2317	164
**Cost of index hospitalization **mean (SD)	$16,981 ($20,474)	$56,024 ($33,207)	$26,254 ($21,089)	$25,022 ($24,857)	$12,052 ($15,152)	$6910 ($7,585)	$15,278 ($17,974)	$12,365 ($12,667)	$6699 ($6,447)	$16,269 ($19,831)
**LOS, days **mean (SD)	4.36 (3.06)	9.23 (3.65)	4.08 (2.41)	4.68 (3.00)	5.59 (3.59)	2.71 (1.40)	3.82 (2.67)	5.44 (3.37)	3.78 (2.83)	6.99 (5.37)

CABG = coronary artery bypass graft; MI = myocardial infarction; TIA = transient ischemic attack; CVA = cerebrovascular accidents; PVD = peripheral vascular disease; LOS = length of stay

Patients could be classified into >1 event type

Mean overall costs for care in the first follow-up year were $16,582 (SD = $34,425; median = $7408) (Table 4). Overall mean follow-up costs increased to $26,644 at 2-year follow-up and to $34,131 at 3-year follow-up.

**Table 4:** Unadjusted costs for CV-event and matched control groups at 1-, 2-, and 3-year follow-up

	Pre-Index	Year 1		Years 1-2		Years 1-3	
	CV-Event (n = 29,688)	CV-Event (n = 29,688)	Control (n = 29,688)	CV-Event (n = 11,620)	Control (n = 11,620)	CV-Event (n = 3853)	Control (n = 3853)
	mean (SD)	mean (SD)	mean (SD)	mean (SD)	mean (SD)	mean (SD)	mean (SD)
Resources Used	median	median	median	median	median	median	median
CV-related medications	$285 ($619)	$813 ($1051)	$180 ($493)	$1676 ($1885)	$394 ($958)	$2530 ($2579)	$662 ($1426)
	$0	$471	$0	$1,121	$0	$1757	$0
All other medications	$1135 ($4511)	$1917 ($5936)	$588 ($2423)	$3687 ($10,272)	$1273 ($4558)	$5064 ($11,456)	$1921 ($4932)
	$149	$756	$20	$1,487	$139	$2166	$399
Emergency room	$186 ($728)	$390 ($1593)	$68 ($447)	$704 ($2086)	$134 ($601)	$1113 ($2734)	$217 ($735)
	$0	$0	$0	$0	$0	$96	$0
Physician office visit	$637 ($2442)	$1,248 ($3409)	$398 ($907)	$2145 ($6820)	$849 ($1550)	$2726 ($5710)	$1208 ($2016)
	$301	$725	$178	$1335	$459	$1836	$719
Imaging tests	$397 ($1624)	$858 ($2130)	$232 ($1252)	$1384 ($3125)	$435 ($1680)	$1616 ($2600)	$551 ($1627)
	$12	$193	$0	$679	$40	$903	$91
Laboratory procedures	$187 ($628)	$356 ($1119)	$118 ($335)	$630 ($1810)	$256 ($607)	$783 ($1541)	$357 ($835)
	$38	$144	$3	$317	$96	$451	$169
All other outpatient services	$1384 ($5070)	$3692 ($9745)	$781 ($3125)	$6051 ($14,075)	$1640 ($6722)	$7826 ($17,797)	$2214 ($5018)
	$127	$1321	$22	$2644	$239	$3600	$594
Hospitalization	$912 ($7120)	$7308($27,680)	$424 ($4258)	$10,367 ($35,024)	$800 ($5082)	$12,473 ($34,671)	$986 ($5488)
	$0	$0	$0	$0	$0	$0	$0
Total Care	$5124 ($13,465)	$16,582 ($34,425)	$2790 ($7837)	$26,644 ($48,086)	$5782 ($12,807)	$34,131 ($51,331)	$8117 ($12,634)
	$1917	$7408	$856	$13,761	$2622	$19,194	$4528

**Figure 2:**
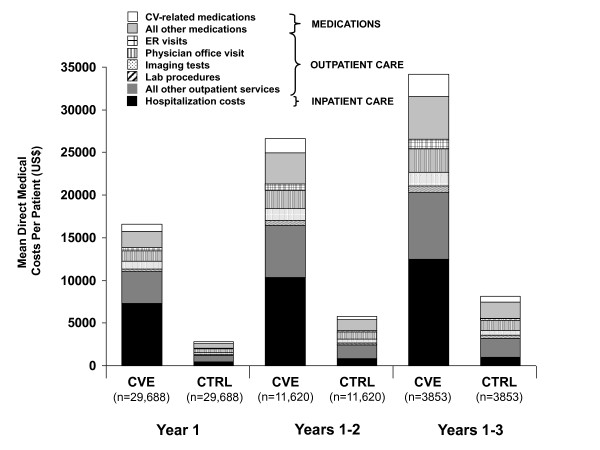
**Unadjusted cost of total medical care in the follow-up period, among (matched) CV-event (CVE) and control - (CTRL) subjects**.

As expected, analysis of the matched cohort of CV-event versus control subjects found that mean total costs for year-1 follow-up healthcare were much higher for CV-event subjects ($16,582) than for those without CV events ($2790) (Table 4), an excess of $13,792 over matched controls' mean cost. Mean total healthcare costs in the year prior to the index CV event ($5124) were lower than in the year following the CV event, but were still much higher than year-1 costs for matched control subjects without a CV event (Table 4). The gap between costs for CV-event subjects and matched control subjects was even larger when mean total costs were compared at 2-year ($26,644 vs. $5782 per subject, respectively) and 3-year follow-up ($34,131 versus $8117 per subject, respectively) (Table 4, Figure 2). The difference in average costs between CV-event and matched-control subjects was $20,862 after 2 years and $26,014 after 3 years of follow-up.

In the multivariable model of main effects adjusting for demographic and clinical differences between subjects, presence of a CV event was the strongest predictor of total 1-year follow-up costs (P <0.0001) (Table 5). Age, hypertension, hyperlipidemia, and diabetes, were also statistically significant predictors of increased costs. Payer type and geographic region were related to increased cost of CV events in some instances, but plan type and physician specialty had no significant association with follow-up costs.

**Table 5:** Multivariable regression model comparing total costs following the index date

Variable	Parameter Estimate	Standard Error	Chi-Square	P-value
CV event: yes vs. no	1.7546	0.0377	2169.7	<0.0001
				
Hypertension: yes vs. no	0.246	0.0158	241.0	<0.0001
Hyperlipidemia: yes vs. no	0.1425	0.0163	76.4	<0.0001
Diabetes: yes vs. no	0.34	0.0213	254.5	<0.0001
				
18-34 years vs. 65+ years	0.5644	0.1162	23.6	<0.0001
35-44 years vs. 65+ years	0.5521	0.1097	25.3	<0.0001
45-54 years vs. 65+ years	0.7040	0.1083	42.3	<0.0001
55-64 years vs. 65+ years	0.8720	0.1082	65.0	<0.0001
				
Female: yes vs. no	0.1792	0.0143	156. 7	<0.0001
				
Plan type: HMO vs. Consumer-directed	0.0194	0.1470	0.02	0.8948
Plan type: Indemnity vs. Consumer-directed	0.1144	0.1490	0.6	0.4427
Plan type: PPO vs. Consumer-directed	0.1540	0.1469	1.1	0.2943
Plan type: POS vs. Consumer-directed	0.0564	0.1482	0.1	0.7036
Plan type: Other/Unknown vs. Consumer-directed	-0.1365	0.1585	0.7	0.3892
				
Payer type: Medicaid vs. Commercial	0.376	0.0798	22.2	<0.0001
Payer type: Medicare Risk vs. Commercial	0.6435	0.1068	36.3	<0.0001
Payer type: Self-insured vs. Commercial	-0.2372	0.0346	47.1	<0.0001
Payer type: Other/Unknown vs. Commercial	-0.0627	0.0317	3.91	0.0481
				
Physician specialty: Internal medicine vs. FP/GP	0.0242	0.0538	0.2	0.6529
Physician specialty: Cardiologist vs. FP/GP	0.0857	0.0445	3.7	0.054
Physician specialty: Other vs. FP/GP	0.0128	0.045	0.1	0.7752
Physician specialty: Unknown vs. FP/GP	0.0587	0.0555	1.1	0.2899
				
Geographic region: Northeast vs. West	-0.107	0.0316	11.4	0.0007
Geographic region: Midwest vs. West	-0.0077	0.0292	0.1	0.7924
Geographic region: South vs. West	-0.106	0.0296	12.8	0.0003

HMO = Health Maintenance Organization; PPO = Preferred Provider Organization; POS = Point of Service; FP/GP = family practitioner/general physician

Among the overall CV-event cohort, 4805 (16.1%) patients experienced at least one subsequent CV event leading to hospitalization. The overall mean hospital length of stay was 4.85 days, with CABG having the greatest LOS at 8.87 days per event. The overall mean hospitalization cost for the subsequent CV event was only slightly higher than the overall mean cost per index event ($17,709 vs. $16,981, respectively; data not shown). Differences in mean costs were generally within $3000 or less of each other, with the largest absolute differences occurring for CABG ($46,236 recurrent vs. $56,024 initial) and other CV procedures ($19,023 recurrent vs. $26,254 initial).

## Discussion and Conclusions

In this retrospective claims database analysis, we document the high costs for CV events and follow-up care (for 1 to 3 years) in a commercially-insured US population. In particular, mean healthcare costs among CV-event subjects were substantially higher in post-index years compared to control subjects. Among CV-event subjects, initial hospitalization costs varied widely by type of CV event ranging from $6,699 for a TIA to $56,024 for a CABG.

The 1-year follow-up costs for CV events were almost as high as the initial hospitalization costs, and were much higher at 2- and 3-year follow-up. These data are consistent with previous evidence showing that persons with CVD incur significantly greater direct medical costs than persons without CVD, with the annual lifetime medical cost of treating CVD patients estimated to be 3.4 times greater than for patients without CVD [2,3].

Our data were consistent with previous evidence that has shown that the costs of CV events and associated follow-up care in patients with HTN, DM, and DYS is higher than the overall mean healthcare costs for CV-event subjects without a co-morbidity [4-9]. Previous analyses using the current database have shown that diabetic patients hospitalized for a cardiovascular event incur higher costs for overall cardiovascular care and for most types of individual cardiovascular events than their non-diabetic counterparts [10]. Other analyses have demonstrated that DM patients with cardiovascular co-morbidity had significantly higher total healthcare costs (38.9%; $12,550 vs. $9031), total Emergency Room (ER/hospitalization costs (239.8%; $4845 vs. $1426), total outpatient costs (35.3%; $3956 vs. $2925), and total prescription drug costs (15.1%; $4686 vs. $4071) compared to DM patients without cardiovascular co-morbidity [11]. Similarly, uncontrolled HTN is estimated to result in 39,702 CV events, 8374 CVD deaths, and $964 million in annual direct medical expenditures in the US [12].

The global burden of CVD is increasing and is expected to surpass infectious disease to become the world's leading cause of death and disability by the year 2020 [7]. The American Heart Association estimated the total annual direct medical expenditures for patients with CVD or stroke to be $131.3 billion in 2008 [13], and recent analyses indicate that the actual economic burden to be substantially higher than this [14].

Because of the growing demand for scarce healthcare resources and the introduction of new, more expensive technologies, difficult choices must be made among competing therapeutic options and priorities [15,16]. Economic evaluation provides a means of rationally informing these choices so that the result is an efficient allocation of resources.

Accurate estimates of medical event costs are integral for pharmacoeconomic modeling. However, it is nearly impossible to accurately determine whether the clinical advantages of a novel CV therapy are likely to result in net cost-savings, for example due to reductions in events, procedures or hospital length of stay, if occurrence rates and costs for specific CV events are not known.

Since the claims analyses in this study were retrospective in nature, it is important to highlight some limitations in the interpretation of our findings. Accurate assessment of costs and resource utilization is heavily dependent on the correct allocation of diagnosis, procedure, and medication codes. In order that errors in the assignment of codes were minimal, all patient medical histories were subject to stringent data checks. In addition, only health-care plans that provided information for all members were included in the database, ensuring complete data capture and representative samples. In addition, as the costs reported here are derived from commercial managed care claims data, results may not be generalizable to other populations.

The multivariable model used was adjusted for the presence of DYS, HTN and DM. It is possible that inclusion of other co-morbidities may have affected the results of these analyses., Patients who died were included in our analyses as completed cases; however, patients who were lost to follow-up were excluded. Consequently, mean costs may have been biased towards patients who died soon after hospitalization. These patients may have incurred higher costs, as they may have been sicker and required more expensive treatment. Although our data precludes confirmation of this presumption, the estimates provided from our analysis remain a reasonable contemporary assessment of the costs within the limitations of our data set.

The findings from this study provide important estimates for pharmacoeconomic modeling of cardiovascular events, and are expected to benefit multiple stakeholders, including patients and policy-makers deciding how to most efficiently allocate healthcare resources.

## Competing interests

This study was sponsored by Pfizer Inc. The topic of the study was agreed upon with Pfizer Inc at the outset of the project; however, the authors retained full rights in the design, analysis, interpretation, and publication of the results. RJS was a paid consultant to Pfizer Inc in connection with the development of this manuscript. PSG and RHC are employees of IMS Health, who were paid consultants to Pfizer Inc in the development of this manuscript. LZL is an employee of Pfizer Inc with ownership of stock in Pfizer Inc. Editorial support was provided by Dr John Bilbruck at UBC Scientific Solutions Ltd and Karen Trochlil and Katharine Coyle at IMS Health and was funded by Pfizer Inc.

## Authors' contributions

RHC contributed to the conception, design and co-ordination of the study, and participated in statistical analysis and programming, analysis and interpretation of the data, and development of the manuscript. LZL was involved in the conception and design of the study and participated in the analysis of the data and the development of the manuscript. PSG contributed to the design of the study and was involved in statistical analysis and programming. RJS participated in the analysis and interpretation of the data and was involved in the development of the manuscript. All authors read and approved the final manuscript.

## Pre-publication history

The pre-publication history for this paper can be accessed here:

http://www.biomedcentral.com/1471-2261/11/11/prepub
